# Potential Parasite Transmission in Multi-Host Networks Based on Parasite Sharing

**DOI:** 10.1371/journal.pone.0117909

**Published:** 2015-03-06

**Authors:** Shai Pilosof, Serge Morand, Boris R. Krasnov, Charles L. Nunn

**Affiliations:** 1 Mitrani Department of Desert Ecology, Albert Katz International School for Desert Studies, Jacob Blaustein Institutes for Desert Research, Ben-Gurion University of the Negev, Midreshet Ben-Gurion, Israel; 2 Institut des Sciences de l'Evolution de Montpellier (ISEM), Centre National de la Recherche Scientifique (CNRS), Montpellier, France; 3 Unité de Recherche Animal et Gestion Intégrée des Risques, Centre de Coopération Internationale en Recherche Agronomique pour le Développement, Montpellier, France; 4 Centre d'Infectiologie Christophe Mérieux du Laos (CICML), Ministry of Health of Lao PDR, Vientiane, Lao PDR; 5 Mitrani Department of Desert Ecology, Jacob Blaustein Institutes for Desert Research, Ben-Gurion University of the Negev, Midreshet Ben-Gurion, Israel; 6 Department of Evolutionary Anthropology & Duke Global Health Institute, Duke University, Durham, North Carolina, United States of America; University of Glasgow, UNITED KINGDOM

## Abstract

Epidemiological networks are commonly used to explore dynamics of parasite transmission among individuals in a population of a given host species. However, many parasites infect multiple host species, and thus multi-host networks may offer a better framework for investigating parasite dynamics. We investigated the factors that influence parasite sharing – and thus potential transmission pathways – among rodent hosts in Southeast Asia. We focused on differences between networks of a single host species and networks that involve multiple host species. In host-parasite networks, modularity (the extent to which the network is divided into subgroups of rodents that interact with similar parasites) was higher in the multi-species than in the single-species networks. This suggests that phylogeny affects patterns of parasite sharing, which was confirmed in analyses showing that it predicted affiliation of individuals to modules. We then constructed “potential transmission networks” based on the host-parasite networks, in which edges depict the similarity between a pair of individuals in the parasites they share. The centrality of individuals in these networks differed between multi- and single-species networks, with species identity and individual characteristics influencing their position in the networks. Simulations further revealed that parasite dynamics differed between multi- and single-species networks. We conclude that multi-host networks based on parasite sharing can provide new insights into the potential for transmission among hosts in an ecological community. In addition, the factors that determine the nature of parasite sharing (i.e. structure of the host-parasite network) may impact transmission patterns.

## Introduction

Parasites play a major role in the lives of animals and humans. In attempts to understand the ecological processes leading to infection with a particular parasite, ecologists have investigated the factors influencing the interaction between the host species and the parasite in question. In recent years, the limitations of this “single-host-single-parasite” perspective have become apparent due to the wealth of indirect effects that parasites and hosts exert on each other within a community, and given the recognized importance of understanding cross-species parasite transmission [1–3]. Specifically, considering a multi-host-multi-parasite system is necessary because the dynamics of a parasite in one species can depend on its dynamics in another [4], and because within a host, co-infection can affect the dynamics of the parasites involved [5].

Network analysis offers a new approach to uncover the complexity underlying interactions among multiple hosts and parasites in an ecological community [6]. The biological interactions among hosts and parasites are depicted as a bipartite host-parasite network, in which edges describe infection of hosts by parasites (interactions among hosts or among parasites are not allowed). A network approach elucidates how properties of the whole network emerge from the properties of its nodes, allowing examination of the system at both the node and network levels [7,8].

Typically, the units of analysis in host-parasite networks are species rather than individuals. However, we lose valuable individual-based information by aggregating individual observations into species-averages [9,10]. The loss of individual-level information is especially important in disease ecology because parasite transmission necessarily occurs at an individual level (individuals are infected, rather than species). In addition, within an individual host, co-infection with multiple parasites can determine both infection with subsequent parasites and the transmissibility of parasites to other individuals [4]. The individual level is also important because large variation exists among individuals in characteristics that promote parasite transmission. Indeed, disease outbreaks may be promoted by a small fraction of individuals who are responsible for the majority of transmission events (‘super-spreaders’) [11,12].

Unlike host-parasite networks, epidemiological networks attempt to characterize parasite spread among host individuals of a single species [13–15]. Epidemiological networks are unipartite (contain one set of nodes), with edges representing contact patterns or some other type of individual-based interaction meaningful for transmission of the parasite in question [13]. In studies of a sexually transmitted disease, for example, an epidemiological network may represent mating patterns among individuals in a social group or population. This approach is essentially a single-host species-single-parasite species approach.

Previous work has highlighted the importance of considering multiple hosts and host heterogeneity in studies of parasite transmission [1–3,16–18]. To date, however, this has not been applied in the context of epidemiological networks involving multiple species. Most likely, this absence reflects the challenges of constructing such networks, which requires simultaneous capture or observation of multiple species in the same time and place [13,19]. We argue that it is important to explore multi-host networks, especially for understanding the effects of heterogeneity at two levels: (i) population-level characteristics shared by all members of a species in the sampled population (e.g. niche breadth, sociality, and abundance) and (ii) individual characteristics associated with variation in parasite acquisition, such as variation in age [20], sex [21] or immunocompetence [22]. In contrast, in single-species networks heterogeneity is only a consequence of individual-level characteristics.

**Fig 1:**
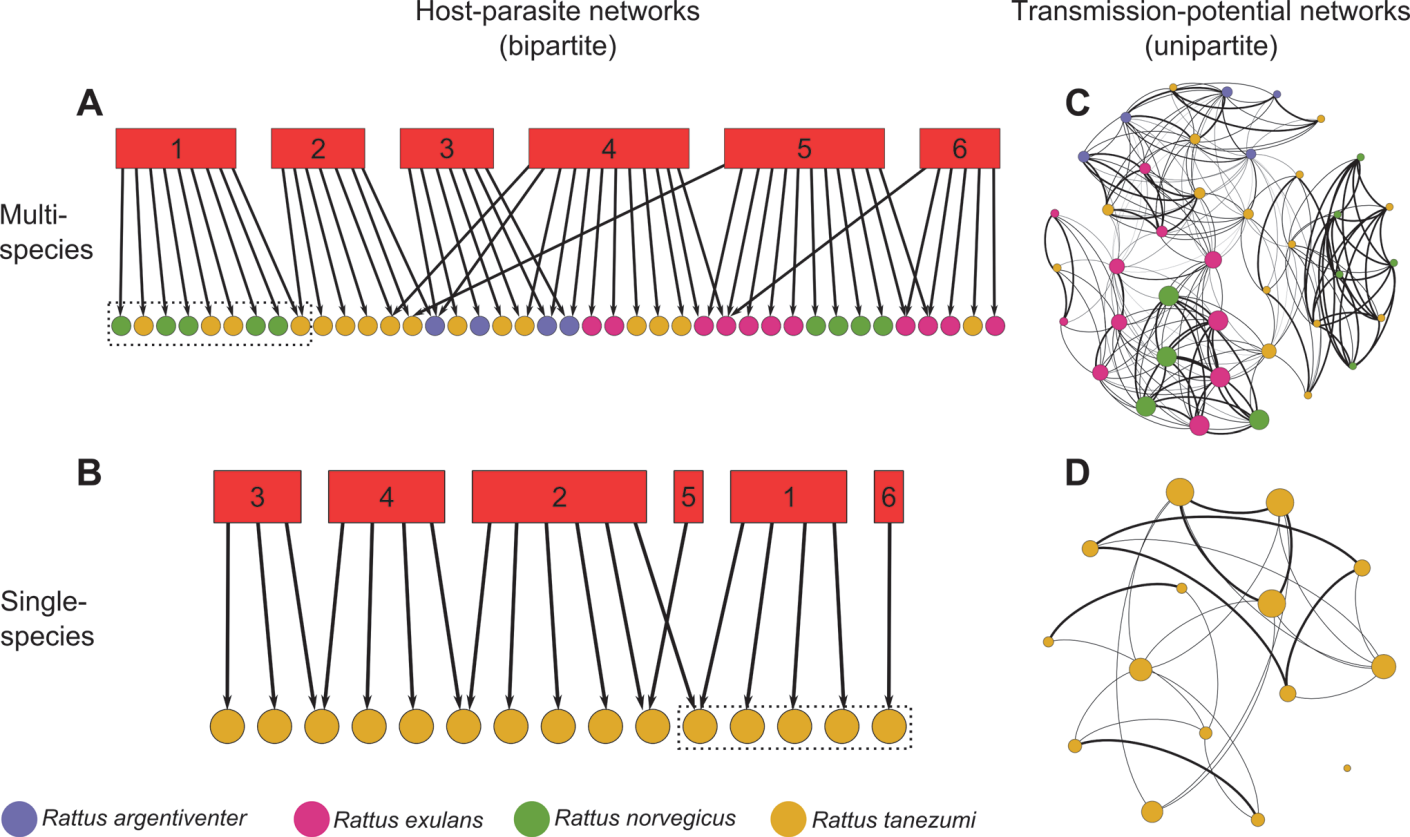
Network types used in this study. Host individuals and parasite species are depicted as circles and squares, respectively, with different colors representing different host species and parasites are represented by numbered red boxes. Networks in **A** and **B** are bipartite networks in which an edge represents infection of a host individual with a parasite species. Examples of modules composed of host individuals are depicted by dashed rectangles. Networks in **C** and **D** are transmission-potential networks created by connecting two individual hosts from the networks in **A** or **B**, respectively, if they share at least one parasite species. The weight of an edge between two individuals is the similarity (beta-diversity) in parasites infecting a pair of individuals, calculated with the Jaccard index (the thicker the edge, the more similar). The size of the nodes is relative to their position in the network, calculated with eigenvalue centrality (larger means more central).

Here, we extend previous studies by investigating multi-host networks using a hybrid approach. First, we examine structural heterogeneity driven by individual- and population-level characteristics in host-parasite bipartite networks composed of individuals of the same and different species. (Fig. 1A,B). Some of these characteristics, such as sex, may represent contact parameters in a typical epidemiological network.

Second, we examine structural heterogeneity and parasite dynamics in unipartite “transmission-potential networks” (TPN) based on parasite sharing. Our general approach is to *project* the bipartite host-parasite networks to unipartite networks by connecting two host individuals if they share at least one parasite species (Fig. 1C,D) [23,24]. Although using networks based on parasite sharing can yield important insights into the relationship between network structure and possible transmission patterns [23,24], a complete picture of the system requires an analysis of both the host-parasite and the (projected) transmission networks, because the structure of the latter is a direct result of the structure of the former. In addition, the effect of the inclusion of multiple hosts in such networks on parasite dynamics has not been investigated.

We used data on south-east Asian rodents and their gastrointestinal helminth parasites. To assess the value of including multiple species, we compared multi-versus single-species individual-based networks (Fig. 1) to test two hypotheses. First, we hypothesized that the difference in individual- and population-level sources of heterogeneity translates to structural differences between multi- and single-species networks. We examined this hypothesis using modularity, which is a network property crucial to the ecology and evolution of hosts and parasites [25–27]. Generally speaking, modular networks (either unipartite or bipartite) are characterized by distinct network subgroups (modules) composed of nodes interacting preferentially among themselves than with other nodes in the network. In host-parasite networks constructed at the species level, modules may be composed of host species more closely related phylogenetically [25,26], partly because closely related species are closer in characteristics that determine compatibility between hosts and parasites. In individual-based networks, we expect the same phenomenon: the tendency for a pair of individual hosts to occur in the same module (i.e. to be infected by similar parasites) should increase with phylogenetic proximity and as similarity in characteristics between individuals increases.

Second, at the node level, we hypothesized that the factors that affect the roles that individuals play in potential parasite transmission differ between multi- and single-species networks. This role can be quantified by indices of centrality where a central node is one that is highly connected to and reachable from other nodes. In epidemiology, central individuals can be considered as super-spreaders [13,28], and the same may be true in transmission networks based on parasite sharing. We therefore used node centrality to capture a node's potential to spread parasites relative to other nodes in the network. We expected that species identity is a strong factor influencing centrality because some host species have been shown to be more central than others [29,30].

Finally, to link network structure to parasite dynamics and to put our results in an applied context, we simulated the spread of a novel parasite in the transmission-potential networks.

## Results

### Data set

We used data on 104 individual rodents from six species and their helminths parasites from three human-disturbed localities: Buriram (14°89’N; 103°01’E; Thailand), Mondolkiri (12°12’N; 106°89’ E; Cambodia) and Sihanouk (10°71’N; 103°82’E; Cambodia). Our data set was unique as it allowed us to test our hypotheses in three different communities with similar characteristics and contained information on individual- and population-level characteristics as well as parasitism. Rodents were parasitized by 13 taxa of gastro-intestinal helminths, identified to species level (see S1 Methods for details on the study system). For each locality, we built one multi-species unweighted (the values of the edges were 1 or 0) bipartite host-parasite network in which edges depicted infection and were drawn between parasite species and host individuals (Fig. 1A). We then extracted from that network smaller single-species networks in which individual hosts belonged to the same species (Fig. 1B).

We selected five individual characteristics potentially associated with variation in parasite acquisition: sex, age (adult versus young), immunocompetence, body mass and habitat in which an animal was caught (forest, lowland/upland agriculture and settlement) because the likelihood of exposure to particular parasites varies with habitat. As a proxy for immunocompetence, we used the ratio of spleen mass to body mass (RSM), with a larger ratio indicating higher immunocompetence [31]. We considered heterogeneity at the rodent species level by using either phylogenetic distance between species or a factor with species identities as levels.

### Network modularity in host-parasite networks

In the host-parasite networks (Fig. 1A,B), we identified modules composed of rodents (rather than rodents and parasites) that interact with similar parasites with a simulated annealing algorithm that finds the maximization of the modularity function *M* (see [32,33] for a detailed explanation). To ensure that the observed modularity is a true biological pattern rather than a result of a random process, we tested for significance of *M* by comparing the observed value to those derived from 1000 random networks constructed with a probabilistic null model [34] that assumed that the probability of drawing an edge between a rodent individual and a parasite species is proportional to the susceptibility of the rodent to parasites (i.e. it considers the number of parasites infecting the individual) and to the infection potential of the parasite (i.e. it considers the number of individuals infected by the parasite).

For host-parasite networks with >10 nodes, all but one single-species network (*Bandicota savilei* in Buriram) were significantly modular (Table 1). The three multi-species networks were evenly fragmented with four modules in each but modularity (*M*) of the multi-species networks of Buriram and Sihanouk was ≈1.8 times stronger than in Mondolkiri. Modularity was higher in the multi-species than in the single-species networks in Buriram and Sihanouk, but not in Mondolkiri (Table 1). Differences in *M* between multi- and single-species networks were generally not a result of differences between network size or connectance—the proportion of realized interactions out of all possible ones (S1 Methods).

**Table 1:** Information on multi- and single-host bipartite networks.

	**# Individuals**	**# Helminth taxa**	**Parasite richness (range, mean ± SD)**	**C**	***M* (Number of modules)**
BURIRAM					
Multi-species	27	10	1–3, 1.63±0.63	16.3%	M_SA_ = 0.53 (4) *** M_T_ = 0.22
Bandicota savilei	15	7	1–3, 1.93±0.59	27.6%	0.24 (5)
Mus cervicolor	6	4	1–2, 1.33±0.52	33.3%	
Rattus exulans	6	3	1–2, 1.17±0.41	38.9%	
MONDOLKIRI					
Multi-species	37	8	1–4, 1.95±0.85	24.3%	M_SA_ = 0.29 (4) *** M_T_ = 0.06
Bandicota savilei	23	7	1–4, 2.13±0.87	30.4%	0.24 (3) *
Rattus tanezumi	14	6	1–3, 1.64±0.74	27.4%	0.33 (3) *
SIHANOUK					
Multi-species	40	6	1–3, 1.32±0.57	22.1%	M_SA_ = 0.54 (4) *** M_T_ = 0.26
Rattus argentiventer	5	3	1–3, 1.8±0.84	60%	
Rattus exulans	11	3	1–3, 1.45±0.69	48.5%	0.25 (3) **
Rattus norvegicus	9	2	1, 1±0	50%	
Rattus tanezumi	15	6	1–2, 1.27±0.46	21.1%	0.52 (4) ***

Parasite richness is the number of helminth taxa infecting an individual rodent. M_SA_—modularity obtained through simulated annealing; M_T_—modularity obtained by pre-determining module composition by taxonomy (one module per species); C—network connectance—is the number of realized interactions divided by the number of possible ones. Statistical significance of modularity

* P < 0.05

** P < 0.01

*** P < 0.001.

**Fig 2:**
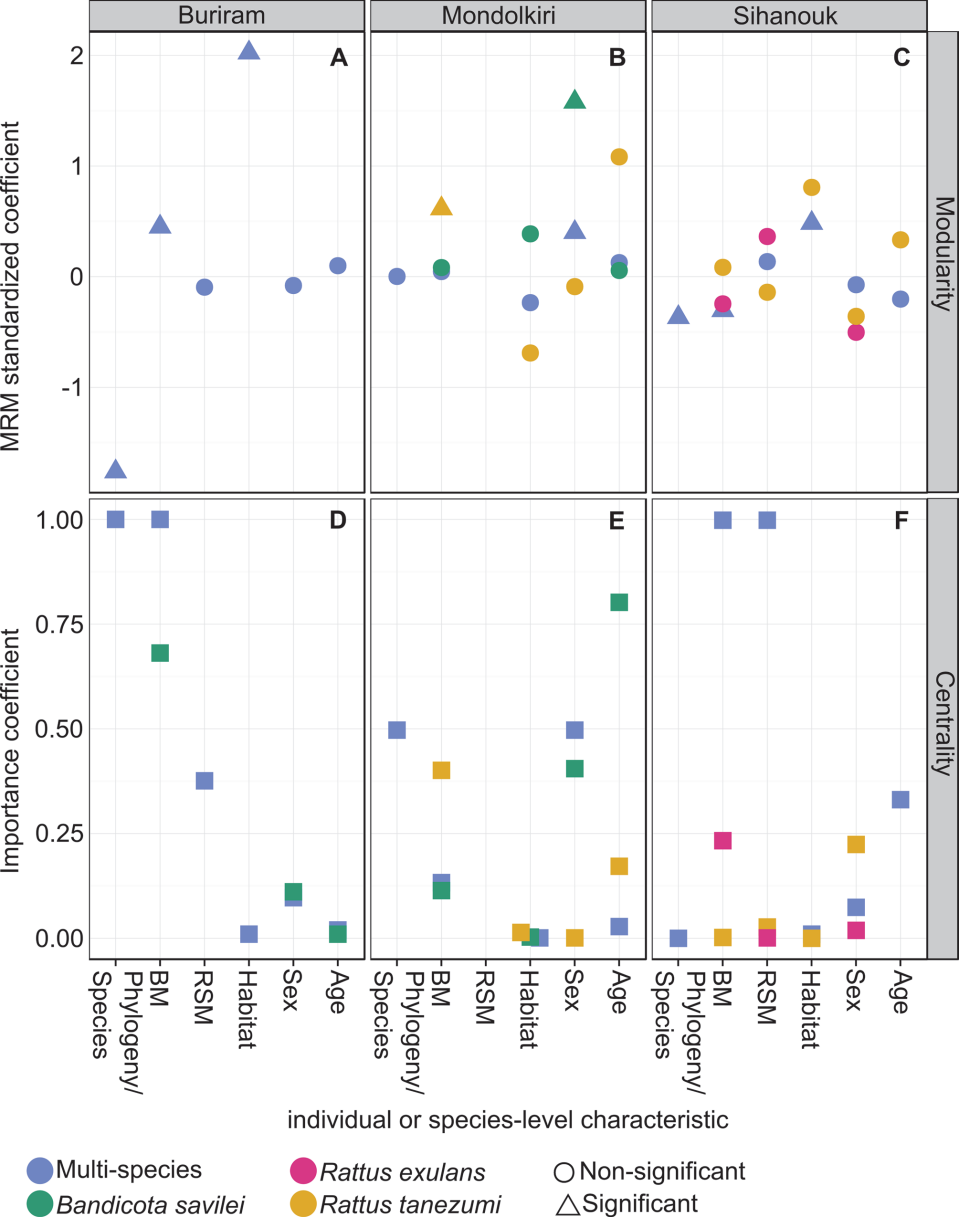
Differences between multi- and single-species networks. Differences are in characteristics that determine co-occurrence in modules and centrality of individuals (rows) for three localities (columns). (**A-C**) The *z*-score-standardized coefficients of a multiple-regression on matrices procedure. (**D-F**) The importance of coefficients calculated from a multi-model inference procedure as the sum of model weights across all the models in which the coefficient appears (see S1 Table). In **A-C** ‘phylogeny’ is the taxonomic distance between two individuals. In **D-F** ‘species’ is a factor depicting rodent species identity. BM—body mass; RSM—relative spleen mass to body mass. Note that: (i) in Buriram the single-species network was not analyzed because it was not statistically significantly modular; (ii) RSM was not included in the analyses in Mondolkiri due to an excess of missing cases; (iii) Statistical significance is relevant only for **A-C**.

After determining the modular structure of the networks, we tested the effect of individual- and population-level characteristics on the affiliation of individuals to modules (module composition) with a logistic multiple regression on distance matrices (MRM), following [26] (S1 Methods). The phylogenetic distance between individuals was a significant predictor of affiliation to modules in Buriram and Sihanouk (but not in Mondolkiri): the closer two individuals were phylogenetically, the more likely that they occurred in the same module (Fig. 2A,C). Other characteristics like habitat and body mass were also significant predictors of the affiliation of individuals to modules in the multi-species networks of Buriram (Fig. 2A) and Sihanouk (Fig. 2C). The importance of individual-level characteristics became even more evident when we pre-determined module composition by taxonomy. In that analysis, the value of *M* was much lower than when obtained through simulated annealing (Table 1).

When considering only single-species networks, none of the characteristics that we proposed was a significant predictor of module affiliation (except sex in *B*. *savilei* and body mass in *Rattus tanezumi* in Mondolkiri). Looking more closely at the standardized coefficients, a large difference between the coefficient of the multi-species network and that of a single-species network indicates that the effect of the characteristic on the probability that two individuals will co-occur in the same module changes upon inclusion of other species. In Mondolkiri, for example, the effect of sex was stronger when considering only *B*. *savilei* than when considering all species (Fig. 2B). In contrast, in Sihanouk sex had a relatively constant effect when considering all species and for each species in particular (Fig. 2C).

### Centrality in transmission networks

To examine the factors that affect the roles that individuals play in potential parasite transmission, we projected each of our single- and multi-species bipartite host-parasite networks to unipartite TPNs by connecting two individual hosts in the unipartite network if they shared at least one parasite species in the bipartite network (Fig. 1). In our parasite-sharing transmission networks, the meaning of an edge is not equivalent to networks constructed based on contact patterns. Whereas contact networks represent potential for transmission based on co-occurrence in space and time, we follow others [23,24] by assuming that edges in our transmission networks depict the potential for transmission between a pair of individuals of the same or different host species based on ecological and physiological characteristics that promote parasite sharing. By transmission potential, we mean the likelihood that a given individual will infect another individual, relative to other individuals in the network, based on observed parasite sharing. Thus, connected individuals form part of the same transmission chain [24].

We calculated edge weights using the Jaccard index, which is a measure of beta diversity [35], assuming a positive relationship between the *similarity* in parasite infections shared by a pair of individuals and the likelihood that a novel parasite would infect them both. Thus, an edge received its minimum value of 0 when the pair of individuals did not share any parasite and its maximum value of 1 when the pair of individuals were parasitized by the exact same species.

We used eigenvalue centrality (EC) to quantify the role of a node in terms of promoting parasite transmission. With EC, a node's importance is increased when it has more connections to other nodes that are themselves important [8]; EC thus enables quantification of the transmission potential of an individual [15,36]. We examined the effect of individual- and species-level characteristics on EC with a set of linear models for each of the multi-species TPNs and for single-species TPNs with >10 individuals. Results of this model selection indicated that species identity was a strong determinant of the position of individuals in the multi-species TPN in Buriram but less so in Mondolkiri. In Sihanouk, species identity did not predict centrality at all (Fig. 2D-F; see S1 Table for detailed results of model selection). This indicated that the effect of species identity on centrality is strongly network-dependent. We found differences among the multi-species networks in the importance of characteristics (Fig. 2D-F). For example, in Sihanouk the body mass of individuals was an important predictor of centrality whereas in Mondolkiri sex was important. As with modularity, we found inconsistencies between the multi- and single-species networks in the importance of individual-level characteristics that affect centrality within a locality. For example, in Mondolkiri, sex was an important predictor of centrality in the multi-species network and in the single-species network of *B*. *savilei* but not in that of *R*. *tanezumi*. In contrast, age was a poor predictor of centrality in the multi-species network but a strong one for *B*. *savilei* (Fig. 2E).

**Fig 3:**
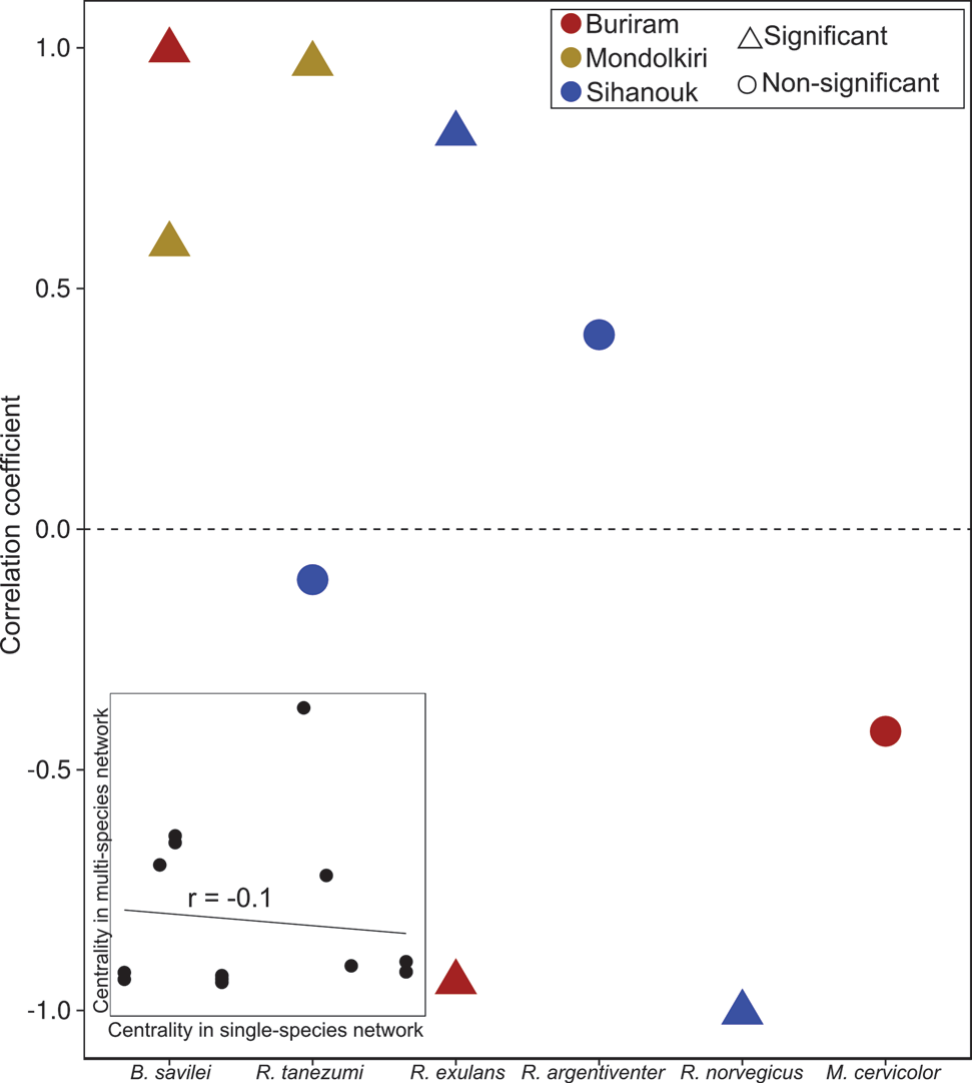
Centrality in single-versus multi-species networks. Data points depict Pearson correlation coefficients between the rescaled eigenvalue centrality of individuals of a particular species in the single-species network and in the multi-species network. Inset: an example for *Rattus tanezumi* in Sihanouk. Note that in the inset data points represent individuals, with some overlapping data points (i.e. individuals with identical centrality values).

The position of specific individuals in the single-species networks in relation to their respective multi-species networks was maintained for some host species but not for others as indicated by a correlation between the centrality of individuals in a particular single-species network and their centrality in the corresponding multi-species network (Fig. 3). For example, individuals of *Rattus norvegicus* in Sihanouk, which were very central in the multi-species network (high centrality), were peripheral (low centrality) in the single-species network, as indicated by a negative correlation coefficient.

### Parasite transmission dynamics

To link network structure to parasite dynamics and to put our results in an applied context, we simulated the spread of a novel parasite across the TPNs with a SI (susceptible-infected) epidemiological model in which an individual can be either susceptible to the parasite or infected and thus infectious. At the start of each simulation, one rodent individual was randomly selected to be infected. In subsequent time steps, the parasite was allowed to spread across rodents in the network. The probability of parasite transmission from rodent individual *i* to its neighbor *j* in the next time step was calculated as *P_i→j_* = 1 −(1 − *θ*)*^w_ij_^*, where *w*_*ij*_ is the edge weight assuming that a stronger weight leads to an increased infection probability [33]. The parameter *θ* is a fixed infection probability, characteristic to the novel parasite in the host species to which the parasite is spreading [33]. We set *θ_s_* = 0.02 in the single-species TPNs.

In the multi-species TPNs, we set *θ_m_* = 0.02 ⋅ *β_nk_*, where *β*_*nk*_ is the Jaccard index of shared parasites between species *n* and *k* [37,38], assuming that the infection probability of individuals of different species was linearly proportional to the similarity in parasites shared by the host species [37]. Because *β*_*nk*_ ranges between 0 (no shared parasites) and 1 (complete sharing), infection probability was at a maximum of 0.02 (the value of *θ*_*s*_) for individuals from species with completely overlapping parasite communities (same species) and 0 for individuals from species with no shared parasites. Our model thus allowed us to account for parasite sharing both at the individual and the species levels and took phylogeny into account because closely related species tend to share more parasites [37,38]. It also considered the relative contribution of within-species transmission to cross-species transmission and the common assumption of multi-host models that within-species transmission is greater than between-species transmission [1,2,16].

To separate the effect of network structure from that of differential infection probability, we also repeated these analyses with a fixed value of *θ_m_* = 0.02 in the multi-species networks, simulating a constant infection probability among all species.

The time steps required to infect all individuals in the network was used as a measure of parasite spread efficiency that we defined as time to global infection (TGI), sensu [33]. Our final response variable was the average value of TGI ($\bar{TGI}$) obtained from simulations of 250 sub-TPNs which controlled for the size and connectance of the compared networks.

**Fig 4:**
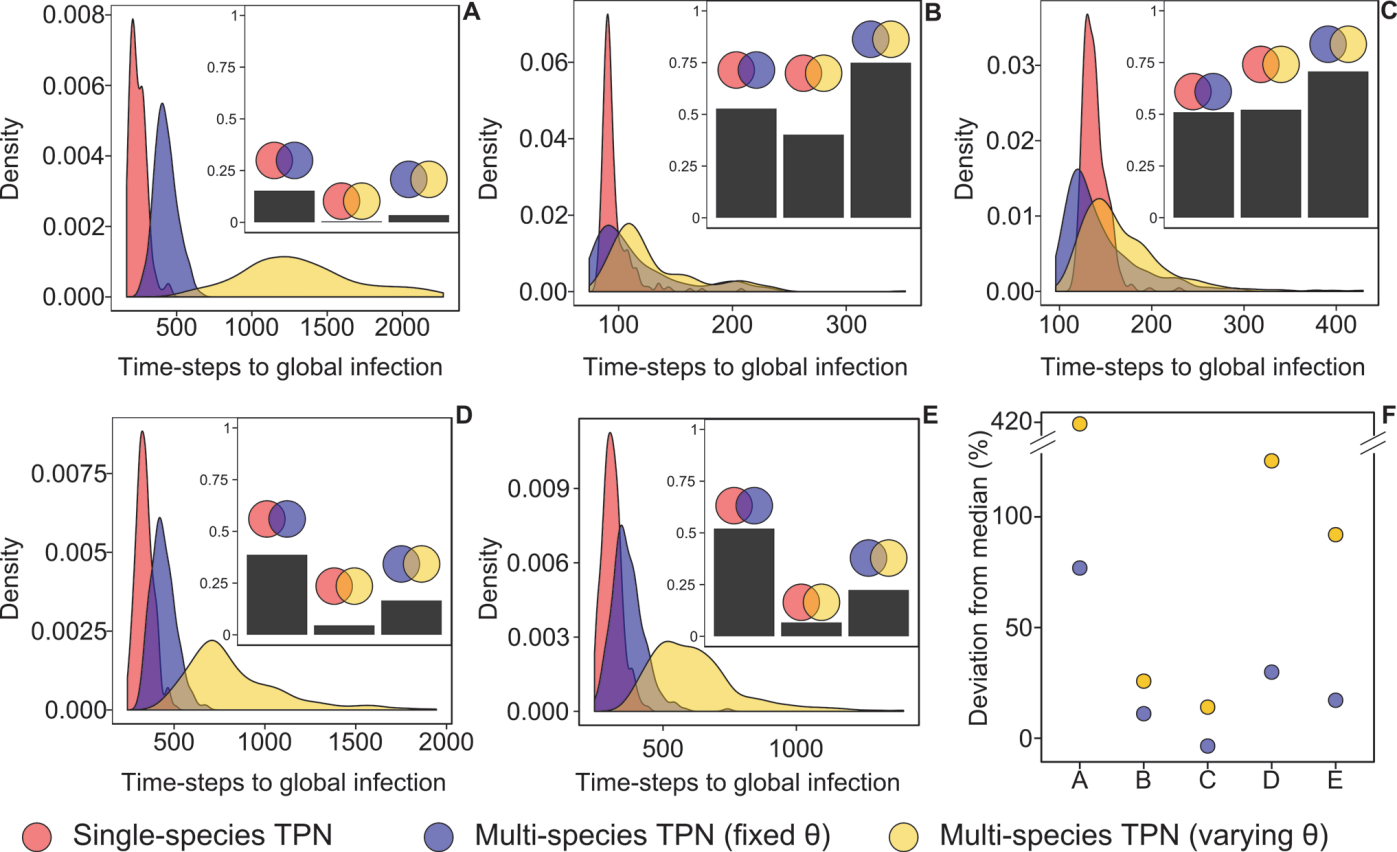
Density plots depicting the distribution of time to global infection (TGI) for transmission-potential networks (TPNs). TPNs were of equal size and connectance under three conditions: single-species, multi-species with *θ*_*m*_ (infection probability) varying among species and multi-species with fixed *θ*_*m*_ = 0.02. Each panel describes a comparison of a given single-species network to multi-species networks in a given locality: (**A**) *Bandicota savilei* in Buriram; (**B**) and (**C**) *B*. *savilei* and *Rattus tanezumi* in Mondolkiri, respectively; (**D**) and (**E**) *R*. *exulans* and *R*. *tanezumi* in Sihanouk, respectively. Plots skewed to the left indicate a faster infection and curve height is indicative of the probability that global infection occurs at a certain pace. Insets in **A-E** show the overlap between two curves (depicted by the colored circle above the bar) calculated as the integral of the area common to both curves. (**F**) The deviation in median $\bar{TGI}$ of the multi-species networks with fixed (blue circles) or non-fixed (yellow circles) *θ*_*m*_ from median $\bar{TGI}$ of the single-species network. The deviation was calculated for each of the panels A-E. Small overlap and a greater deviation of the median between the single-species and the multi-species plots mean that the inclusion of other species changes the velocity of parasite spread.

Overall we made five comparisons of a single-species to a multi-species TPN: *B*. *savilei* in Buriram, *B*. *savilei* and *R*. *tanezumi* in Mondolkiri, and *R*. *exulans* and *R*. *tanezumi* in Sihanouk (Fig. 4). In each of these comparisons, we had three density plots, describing the $\bar{TGI}$ distribution, corresponding to single- and multi-sub-TPNs with fixed and varying *θ*_*m*_, the infection probability. The density plots significantly differed among each other in all five comparisons made: (Kolmogorv-Smirnov test, P < 0.0001 in all cases). This emphasized the differences in parasite dynamics between single- and multi-species networks.

Looking more closely, In Buriram and Sihanouk (Fig. 4A,D,E) infection occurred at a slower pace in the multi-species than in the single-species TPNs when infection probability differed among species (*θ*_*m*_ was not fixed), as indicated by large deviation of the median $\bar{TGI}$ (yellow circles in Fig 4F). This deviation was much lower when infection probability was equal for all species (i.e. fixed *θ*_*m*_; blue circles in Fig. 4F). Continuing this line of evidence for Buriram and Sihanouk, dynamics were considerably different between single-species and multi-species TPNs with a varying infection probability, as indicted by a very low overlap index. The similarity in dynamics increased, but still remained <50%, when infection probability was fixed (Insets in Fig. 4A,D,E). This indicated that differences in network structure between single- and multi-species networks played an important role in determining dynamics. In contrast, in Mondolkiri, the deviation from the median was not high (Fig. 4B,C,F) and the overlap in the plots between the single and multi-species scenarios was similar regardless of a fixed infection probability (insets in Fig. 4B,C). These differences between the localities indicated that dynamics, just as modularity and centrality, are context- (and therefore network-) dependent.

For the multi-species scenario, a large overlap between the density plots of the fixed/non-fixed *θ*_*m*_ indicates a small effect of species composition on dynamics. This was evident in Mondolkiri (but not in Buriram and Sihanouk; Fig. 4), with an overlap of almost 75%, emanating from the fact that *B*. *savilei* and *R*. *tanezumi*, the only two species present in Mondolkiri, are closely related and thus inter-specific difference had little influence on *θ*_*m*_. In Buriram and Sihanouk there were more species, which were also more distant, creating larger differences in *θ*_*m*_ expressed as a lower overlap (Fig. 4A,D,E).

## Discussion

A primary aim of disease ecology is to understand host-parasite interactions and parasite spread in a particular environment [39]. Using network analysis based on parasite sharing we find that these two processes are intertwined. In addition, our single-versus multi-species comparisons show that considering heterogeneity at both the individual and the species levels gives a more complete view of the system. Our data and associated results represent just one example of projecting networks based on parasite sharing; results for other systems may differ. Thus, we devote much of what follows to consider the broader advantages, assumptions and limitations of using parasite sharing as a method for constructing multi-species networks.

### Single-species versus multi-species networks

We found that the structure of bipartite individual-based host-parasite networks differed between multi- and single-species networks, partially supporting our first hypothesis that predicted stronger modularity in multi-species networks. This indicates that species-level characteristics shape the structure of individual-based host-parasite networks. Individual heterogeneity in some characteristics had similar effects in both multi-and single-species networks, while other characteristics had different effects in the different networks. When scaling up from individual to species-level networks, this may affect the structure of the species-level network [9]. In addition, differences between localities point to the context-dependence of the network itself.

At the node level, the difference between single- and multi-species networks was more striking, indicating that the potential role that individuals play in parasite transmission is a function of both sources of heterogeneity. Previous studies have emphasized the importance of transmission heterogeneity and the identification of super-spreaders [12,28]. Our results suggest that another source of heterogeneity involves differences among species. In support, individuals that were more central in the single-species networks were generally also more central in the multi-species networks.

### Parasite dynamics in multi-host systems

Host heterogeneity is known to be important for parasite infectiousness [17,18]. For example, the introduction of grey squirrels (*Sciurus carolinensis*) infected with parapoxvirus caused a severe decline in a disease-free population of red squirrels (*Sciurus vulgaris*) in England [40]. Previous efforts to understand parasite dynamics in multi-host systems indicated that the ability of parasites to spread in multi-host systems depends on the relative contribution of within-species to cross-species transmission [1,2,16]. Therefore, dynamics is affected by both individual- and species-level sources of heterogeneity. However, current multi-host models of parasite dynamics assume homogeneously-mixed populations. Here, we made a first attempt to quantify dynamics in a multiple host species transmission network model based on parasite sharing. This is a natural next step to the study of VanderWaal et al. [24], which identified individuals and species that are key to transmission in such networks but did not examine transmission *per se*.

Our simulations clearly showed that in networks of the same size and connectance, the dynamics of parasite transmission differs between single-species and multi-host networks. This result was not only due to the relative contribution of within-species vs. cross-species transmission but also due to network structure because it was maintained even when assuming that the parasite infects different species with the same infection probability. Studies that consider transmission only within a single species, as is common in current network models, may thus incorrectly estimate the dynamics of parasite spread, in line with previous studies that demonstrated a dependence of infection probability upon species richness in the community under certain conditions [17,41]. This aspect provides a rich area for future studies of wildlife networks because incorporation of several host species may have several effects on the system, depending on the particular dynamics of the pathogen in each of the species [17] and species characteristics related to their potential to encourage transmission [18].

Interestingly, the process of infection was context-dependent, indicating that different networks may be affected by different processes even when having similar species composition. Therefore, a host species which may be central for disease transmission in one site may be less important in a different site, depending on the ecological context and species community. This finding is crucial for designing adequate control plans.

### The use of transmission-potential networks

Using network models to study parasite dynamics in multi-host systems is advantageous because individuals of different species may not be homogeneously mixed, as is commonly assumed (e.g. [2,40]). This effect can be captured by using parasite sharing as a predictor for parasite spread in TPNs. At the interspecific host level, parasites may be shared through processes occurring at different time scales: cross-species transmission (ecological time scales) and co-inheritance (evolutionary time scales). On the other hand, it can be argued that the mechanisms underlying parasite sharing at the host level may be irrelevant for TPNs because once two individuals of different species share at least one parasite, it is clear that they possess similar physiological (e.g. immune response) and ecological (e.g. diet and habitat preferences) characteristics that would most likely allow them to share a novel one.

To date, only one study that we know of has constructed multi-host networks in which edges represent social contacts [42]. Böhm et al. [42] used proximity collars to record intra- and inter-species contact patterns of badgers and cows to obtain insights into inter-specific transmission of bovine tuberculosis. They showed that badgers tended to interact more frequently with cows than with badgers from other social groups. Moreover, those interactions were with cows with a central position in the herd (more connected). Their study thus emphasizes the importance of using a network approach in studies of cross-species disease transmission. Yet, collecting data adequate for constructing multi-host networks in which edges are observed contact patterns is in the vast majority of cases difficult. Using parasite sharing as an alternative method is thus advantageous.

One limitation of this approach is that the inclusion of different parasites may result in different TPNs. However, this is equivalent to obtaining contact networks with different structure by using different methods (e.g. capture-recapture vs. radio-tracking; [19]). Ideally, the most complete set of parasites should be included in the analysis. A second limitation is that connections between individuals that share a widespread parasite may be over-represented but such an effect can be easily tested for by repeating analyzes with and without the parasite. A third limitation is that TPNs may not represent true individual contacts. However, a recent study by VanderWaal et al. [23] showed that a network based on shared *Escherichia coli* strains co-varied with a network of social contacts in giraffes (*Giraffa camelopardalis*). This finding supports the assumption that transmission pathways based on shared parasites can reflect transmission pathways based on social contacts.

Yet, empirical comparisons between a TPN and a true contact network are needed to further validate this assumption. We are not aware of any data set that includes both individual contacts and a parasite survey in multiple species, but such data could be collected. For example, parasite sharing is possible between primate species sharing a physical space [43]. Following primate groups to document potential transmission edges (e.g. shared space, common food), while simultaneously collecting their feces for a parasite survey would enable construction of a multiple-species network based on shared space use and a TPN.

When working with networks based on parasite sharing, several considerations are important. First, data collection should occur over a rather narrow time window and limited geographical space, depending on the species’ life history and the goal of capturing individual heterogeneity. Second, capture probability should be equal among species—an assumption that in practice is difficult to achieve—but can be controlled for statistically. Finally, transmission mode of parasites included in the host-parasite network should be taken into account. For instance, a TPN derived from a host-parasite network in which parasites are sexually-transmitted is likely to differ from one based on environmental transmission.

The method chosen for quantifying edge weights can also affect the results. For example, VanderWaal et al. [24] created a transmission network of individuals belonging to different ungulate species by connecting a pair of individuals if they shared at least one genetically-determined subtype of *E*. *coli*. Their networks were unweighted—the values of the edges were either 0 (no subtypes shared) or 1 (at least 1 subtype shared)—and thus important information was lost in the projection from the host-subtype to the transmission network (e.g. the number of subtypes shared between individuals). Here, we used quantitative TPNs constructed with one measure of beta diversity (Jaccard index), but other indices are also available [35]. For many of these issues, computer simulations can be usefully applied to investigate options for constructing TPNs from patterns of parasite sharing.

### Applicability and future directions

To date, only a single study has considered a multi-host transmission network, which was also based on parasite sharing via projection (although not explicitly stated) [24]. Here, we show that exploring the original host-parasite network is crucial if we wish to understand the processes underlying the TPN, and we take a first step to understand parasite dynamics in multi-host networks. Further, we identify several future directions that can lead to a better understanding of multi-host networks.

From a modeling perspective, applying other structural indices besides modularity will undoubtedly provide new insights into individual-based multi-species networks. For example, the degree of specialization of individuals and parasite species in the host-parasite network can be measured [44]. From an epidemiological perspective, extensions of the SI model could allow for recovery or an exposed but non-infectious period.

From a disease control perspective, understanding individual-based multi-species networks is essential because it provides a way to model the spread of parasites or pathogens that can switch hosts. Insights from individual-based multi-species models may thus aid disease control efforts by identifying both individuals and species that require greater control, making the efforts more effective [28]. Furthermore, in systems where individual data has already been collected, such as in many parasite surveys (e.g. [45]), TPNs provide an immediate and cost-effective method to preliminarily understand the role of multiple species and individual heterogeneity in disease transmission.

## Conclusion

The use of transmission networks based on parasite sharing can be an advantageous method to understand parasite dynamics in a multi-host context. However, ecological factors that determine the nature of sharing (i.e. structure of the host-parasite network) and the analytical method of network projections should not be overlooked because they can greatly affect the results. By analyzing both host-parasite and transmission networks, insights can be gained from these two perspectives alike for a complete picture of the infection process. In this way novel insights into how parasites are transmitted within a community or assemblage can be gained, thereby opening a new avenue of research at the interface between ecology and epidemiology.

## Materials and Methods

### Network modularity

The simulated annealing algorithm we used identifies modules composed solely of host individuals based on their shared interactions with parasites by maximizing the modularity function *M* [32,33]. The function *M*, intended specifically for bipartite networks, approximates its maximum value of 1 when (i) all host individuals infected by a given parasite species belong to a single module and (ii) the probability of two randomly picked host individuals being infected by the same parasite species is low [32,33].

### Multiple regression on distance matrices

We tested the effect of individual- and population-level characteristics on the affiliation of individuals to modules (module composition) with a logistic multiple regression on distance matrices (MRM), following [26]. A visual description of the method is given in S1 Methods. In each distance matrix in the regression rows and columns depicted rodent individuals and thus matrix cells depicted pairwise differences between individuals. Because pairwise differences are symmetric, only the lower half of the matrix was used. We defined the response matrix **R** as a binary distance matrix where **R**_**ij**_ received a value of 1 if rodents *i* and *j* occurred in the same module and 0 otherwise. Each of the explanatory matrices described pairwise differences between individuals in a certain characteristic (e.g. body mass, sex, etc). For continuous characteristics, the difference was calculated as an absolute difference; for discrete characteristics, 1 was assigned if the two individuals had the same value (e.g. both were males), and 0 if they differed in the characteristic. In the multi-species networks, we included a continuous explanatory variable matrix that contained patristic distances (sum of phylogenetic branch lengths) as a measure of phylogenetic distance between a pair of rodents. The output of the analysis is similar to that of a ‘classic’ logistic regression and includes a list of coefficients and their statistical significance (see [46,47] for details on how statistical significance is calculated in MRM).

We only analyzed networks with >10 individuals and omitted RSM from analyses in Mondokiri because data were missing for seven individuals. We standardized the continuous explanatory variables by converting them to *z*-scores before performing the MRM to avoid effects of different scales. This allowed for a comparison of the relative importance of the predictors [26]. Although an information-theoretic based analysis (as with centrality; see below) was our preferred method, a likelihood function is unavailable for MRM. We thus interpreted our results based on coefficient values and statistical significance.

In addition to the MRM analysis, we also examined the importance of species identity in determining modular structure by calculating *M* (see formula in [32]) in networks that were partitioned into modules composed of individuals of the same species. If species identity is the only, or main, factor affecting module composition we expect the value of *M* to be close to that obtained through simulated annealing.

### Network centrality

We projected each of our single- and multi-species bipartite host-parasite networks to unipartite TPNs by connecting two individual hosts in the unipartite network if they shared at least one parasite species in the bipartite network (Fig. 1). Network projection is commonly used in studies of ecological networks in general and in studies of host-parasites in particular, [29,30,33,48]. Here, our motivation for projecting the host-parasite networks was to create unipartite networks compatible with unipartite epidemiological networks, but that contain multiple hosts. This approach allows for (i) a theoretical comparison with studies of epidemiological networks using single-species networks; (ii) applying similar analytical approaches (e.g. centrality) as in those studies and (iii) modeling parasite transmission across individuals of different hosts.

In projected networks, it is common to set the weight of an edge between two nodes as the number of nodes from the other set they share (e.g. [30]). However, this method may bias the results by the total number of parasites. Instead, we used the Jaccard dissimilarity index, calculated as *a/(a+b+c)*, where *a* is the number of parasites infecting both host individuals, and *b* and *c* are the number of parasites infecting either host individuals. The value of the index scales positively with increase in *a* [35].

We examined the effect of individual- and population-level characteristics on EC with a set of linear models for each of the multi-species TPNs and for single-species TPNs with >10 individuals. Models within a set differed in the characteristics (i.e. sex, age, etc.) they had as explanatory factors, and we included species identity as a factor in our models for multi-species networks (S1 Table). We eliminated factors with no variation (e.g. when all individuals belonged to the same sex), or with an excess of missing data (i.e. RSM in Mondolkiri). For each TPN, we compared models—including a null model with an intercept only—using model probabilities *w* based on AIC corrected for small sample size (AICc), which gives a measure of the plausibility, on a 0 to 1 scale, that a particular model is the best model [49]. We used a measure of coefficient importance, calculated as the sum of *w* across all the models in which the coefficient appears, to quantify the importance of a characteristic in determining EC.

To quantify the effect of the inclusion of several species on the position of individuals in the network we correlated the centrality of individuals in a particular single-species network with their centrality in the corresponding multi-species network using a Pearson correlation for networks with >5 individuals. A positive correlation indicates that individuals with a more central position in the multi-species network are also more central in the single-species network.

### Transmission in multi-host networks

A SI model is particularly suitable for helminths because rodents usually carry the helminthic infection throughout their lives or at least for very long periods. Our model assumed that the novel parasite has similar characteristics to the parasites shared between the individual hosts, and that population densities of the rodent species were equal, although we considered the relative proportion of species abundances in the community (S1 Methods).

We emphasize that the exact value of the infection probability *θ*_*s*_ is unlikely to affect our conclusions because we observe the system from a relative point of view (single vs. multi-species TPNs). Our preliminary sensitivity analysis indeed showed that the results remained qualitatively similar for different values of *θ*_*s*_.

To eliminate the effects of network size and connectance when comparing $\bar{TGI}$ between a multi-species TPN and a single-species TPN within the same locality, we created 250 multi- and single-species sub-TPNs of equal size and connectance derived from the original TPNs within a locality (S1 Methods). We ran the algorithm 250 times per sub-TPN, totaling to 62,500 simulations, and used the average value of each sub-TPN ($\bar{TGI}$) to obtain a distribution of 250 $\bar{TGI}$ values corresponding to 250 sub-TPNs.

We compared the distributions (density plots) of single- and multi-species sub-TPNs using a Kolmogorov-Smirnov test. However, the test only indicates if the $\bar{TGI}$ values originate from the same distribution. It does not pin-point in which way the distributions are different neither does it quantify their degree of similarity. Hence, we also calculated the deviation of the median of the distributions of the multi-species density plots from the median of the single-species density plot as $\left({\tilde{M}}_{m}-{\tilde{M}}_{s}\right)/{\tilde{M}}_{s}$ where ${\tilde{M}}_{m}$ is the median of the density plots of multi-host simulations run with fixed or non-fixed theta and ${\tilde{M}}_{s}$ is the median of the single-species plot. We preferred the median over the mean due to the skewed shape of the distributions (Fig. 4). A large and positive deviation of the median points to a slower rate of infection in the multi-species TPNs.

In addition, to quantify the degree of similarity between the distributions we calculated the integral of the area common to two plots as an index of overlap. The index gets its maximum value of 1 when the two distributions are identical and its minimum value of 0 when the distributions are completely segregated. We expected a difference in infection patterns (shape and position of distributions) between multi-species TPNs and each of the single-species TPNs due to the greater individual heterogeneity in the multi-species networks and the heterogeneity in *θ* in individuals from different species.

### Code and data

Analyses were performed using R (version 3.1.1; [50]) within the Linux environment with aid of the ‘bipartite’ package (version 2.04; [51]). We calculated EC with the ‘evcent’ function from the igraph package (version 0.7.1; [52]). Multi-model inference was done with package MuMIn (version 1.10.5) in R [53]. Modularity analyses were done with software bipartmod (http://seeslab.info/downloads/bipartite-modularity/). MRM analysis was done with a modified version of the ‘MRM’ function from the ‘ecodist’ package (version 1.2.9) in R [54]. We provide the R code and data in S1–S4 Data. Matrix data and original files and code to assemble the phylogenetic tree are also available at Figshare http://dx.doi.org/10.6084/m9.figshare.1210724.

### Ethics statement

All work with animals was approved by the French National Research Agency, project ANR 07 BDIV 012. Animals were treated in accordance with the guidelines of the American Society of Mammalogists and with the European Union legislation (Directive 86/609/EEC). Approval notices for trapping and investigating rodents were given by the Ethical Committee of Mahidol University, Bangkok, Thailand, number 0517.1116/661 based on the validation of the rodents trapping book protocols of CERoPath. Cambodia has no ethics committee overseeing animal experimentation. Additional approval was obtained from the regional Head of Veterinary Service (Hérault, France), for sampling and sacrificing rodents and harvesting rodent tissues (approval no. B 34-169-1). The field studies in all localities (Buriram, Thailand—14°89’N; 103°01’E; Mondolkiri, Cambodia—12°12’N; 106°89’ E and Sihanouk, Cambodia—10°71’N; 103°82’E) did not involve endangered or protected species and none of the rodent species investigated were on the CITES list, nor the Red List (IUCN). Trapping methods included live cage traps. Euthanasia was performed with enflurane or isoflurane and followed guidelines of the American Veterinary Medical Association Council on Research and the Canadian Council on Animal Care.

## Supporting Information

S1 Data Table with individual- and population-level characteristics. (CSV)

S2 Data Matrix depicting infection of individual rodent hosts by parasite species. (CSV)

S3 Data Phylogenetic tree. (TXT)

S4 Data R code of analyses. (R)

S1 Methods Additional description of the methods used in this study. (PDF)

S1 Table Comparison of models used for multi-model inference. (PDF)
