# Supplementary material for: Potential Parasite Transmission in Multi-Host Networks Based on Parasite Sharing
Source: PLoS One. 2015 Mar 6;10(3):e0117909. doi: 10.1371/journal.pone.0117909 (PMC4352066; doi:10.1371/journal.pone.0117909)
Supplement: S1 Methods — (PDF) [file pone.0117909.s005.pdf]

Supporting Methods

1. Study system and data set

We used data from the Community Ecology of Rodents and their Parasites (CERoPath) project. An extensive description of the field and laboratory methodology (including helminths surveys) applied in the project can be found in [1–3] and in the CERoPath website (www.ceropath.org). Briefly, rodents were trapped at three human-disturbed localities: Buriram (14°89'N; 103°01'E; Thailand), Mondolkiri (12°12'N; 106°89' E; Cambodia) and Sihanouk (10°71'N; 103°82'E; Cambodia (Fig. S1). Trapping was conducted during the dry season in November 2008 (Sihanouk and Buriram) and November 2009 (Mondolkiri). At each locality, 30 lines of ten traps, distanced 1 to 5 km from one other were set over four days. The traps were evenly distributed among four habitat types: forest (natural forest and tree plantations); non-flooded upland (shrub, orchards and upland agriculture); lowland flooded areas (rice paddies); and peridomestic locations (houses and immediate surrounding areas).

Helminths survey for each rodent was conducted following [1]. Briefly, trapped rodents were euthanized and dissected. The stomach, small intestine and large intestine were separated and examined for helminth infection under a stereo-microscope. The collected helminths (Table S2) were preserved in 70% alcohol and identified according to general helminth identification keys as referenced in [1,3].

Across the three localities, the three multi-species networks had 27-40 individuals from 2-4 rodent species infected by 6-10 helminth taxa. The single-species networks had 5-23 individuals infected by 2-7 helminths. Helminth richness (number of helminth taxa infecting an individual rodent) ranged between 1 and 4. When averaged across individuals within each

network, mean helminth richness ranged between 1 and 2.13. The prevalence of each helminth in each rodent species is indicated in Table S3.

We built a phylogenetic tree (Fig. S2) based on molecular data of the cytochrome b mitochondrial gene. We compiled cytochrome b sequences from the NCBI gene bank and used a maximum likelihood analysis with the GTR+G+I substitution model of molecular evolution with the aid of the function ‘phymltest’ in the R package ‘ape’ [4]. To ensure that our results were not affected by the way we constructed the tree, we re-ran analyzes with a tree from [5], but that did not include *Mus cervicolor*. The results were qualitatively the same.

Table S2. Information on helminth taxa used in this study. Data are from [3,6]. Helminths in the table are gastrointestinal parasites transmitted via fecal-oral pathways. All helminths were identified to species level (four as unique morpho–species). We included one helminth with direct mode of transmission (*Syphacia muris*) because our preliminary work indicated that removing this helminths did not change our results and this helminths was very common.

ID	Species	Locality	Group	Life cycle (Mode of transmission)	Vector	Zoonotic
1	*Aonchotheca sp*	B	Nematoda	Indirect	Earthworm	
2	*Capillaria sp 1*	M	Nematoda	Indirect	Earthworm	
3	*Echinostoma malayanum*	M	Trematoda	Indirect	Gastropod	+
4	*Eucoleus sp*	M,B	Nematoda	Indirect	Earthworm	
5	*Heterakis spumosa*	M,S	Nematoda	Indirect	Arthropod	
6	*Gongylonema neoplasticum*	S,B	Nematoda	Indirect	Arthropod	
7	*Hymenolepis diminuta*	M,S,B	Cestoda	Indirect	Arthropod	+
8	*Notocotylus loeiensis*	B	Trematoda	Indirect	Gastropod	
9	*Physaloptera ngoci*	M,S,B	Nematoda	Indirect	Arthropod	
10	*Protospiura siamensis*	B	Nematoda	Indirect	Arthropod	
11	*Raillietina sp.*	M,S,B	Cestoda	Indirect	Arthropod	+
12	*Hymenolepis nana*	B	Cestoda	Indirect	Arthropod	+
13	*Syphacia muris*	M,S,B	Nematoda	Direct/ Indirect	Egg ingestion	

B – Buriram; M – Mondolkiri; S – Sihanouk.

Table S3. Prevalence of helminths in rodent species in the three localities. Empty cells indicate that the helminth taxa did not occur in the locality.

	**Buriram**			**Mondolkiri**		**Sihanouk**			
	Bs	*MC*	*Re*	*Bs*	*Rt*	*Ra*	*Re*	*Rn*	*Rt*
Aonchotheca sp	0.07	0.00	0.00						
Capillaria sp 1				0.00	0.29				
Echinostoma malayanum				0.04	0.07				
Eucoleus sp	0.07	0.33	0.00	0.57	0.00				
Herarakis spumosa				0.17	0.00	0.20	0.00	0.00	0.40
Gongylonema neoplasticum	0.00	0.17	0.00			0.00	0.45	0.00	0.07
Hymenolepis diminuta	0.00	0.00	0.67	0.26	0.21	0.00	0.64	0.44	0.07
Notocotylus loeiensis	0.07	0.00	0.00						
Physaloptera ngoci	0.20	0.00	0.00	0.17	0.07	0.80	0.00	0.00	0.20
Protospiura siamensis	0.07	0.17	0.33						
Raillietina sp	0.87	0.00	0.00	0.52	0.71	0.00	0.00	0.56	0.27
Hymenolepis nana	0.00	0.67	0.00						
Syphacia muris	0.60	0.00	0.17	0.39	0.29	0.80	0.36	0.00	0.27

Bs –*Bandicota savilei*; Mc – *Mus cervicolor*; Re – *Rattus exulans*; Rn – *Rattus norvegicus*; Rt – *Rattus tanezumi*.

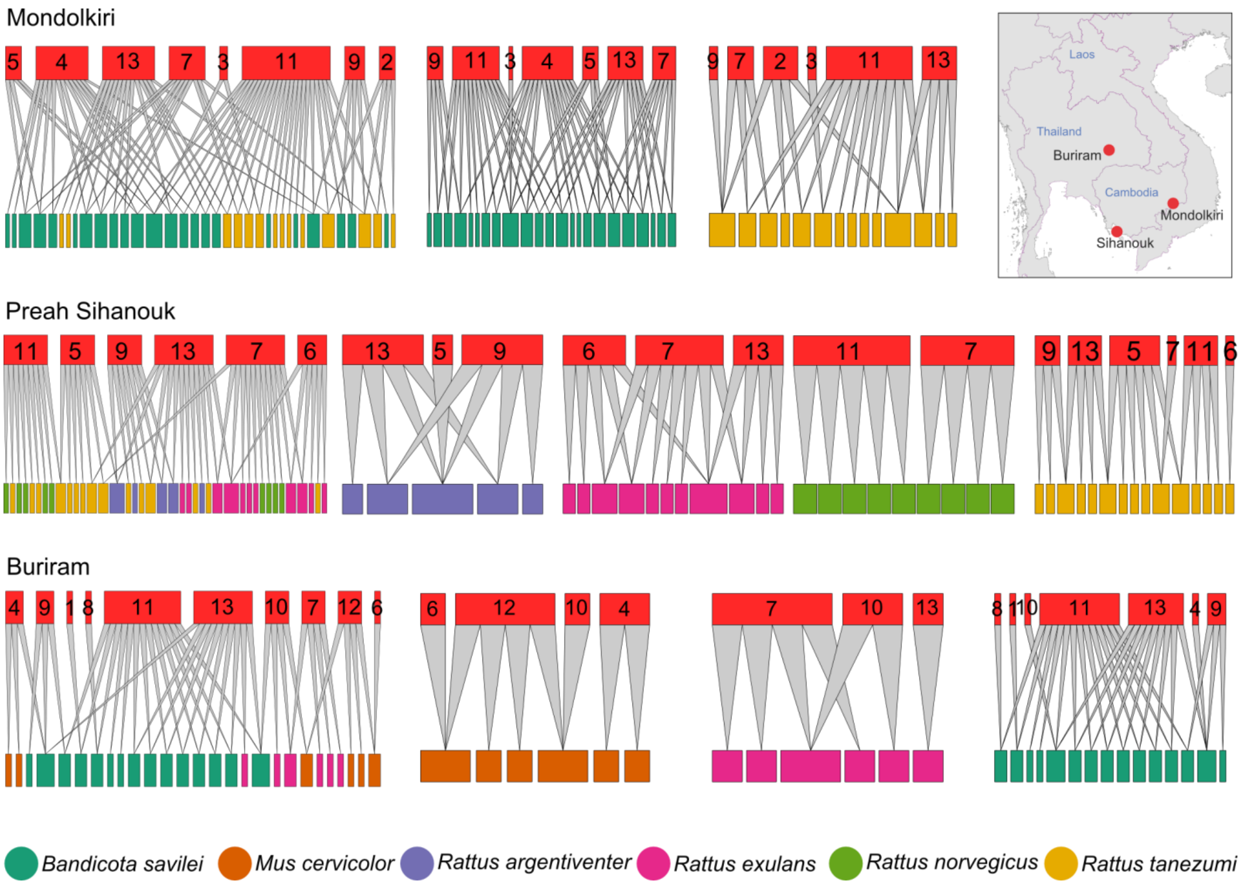

Figure S1. Host-parasite networks at the three localities. The leftmost network at each locality is the multi-species network. In each network helminth taxa (upper nodes) are in red and their ID numbers correspond to Table S2. Lower nodes are individual rodents, and their color represents their species. Width of rectangles is proportional to the number of individuals infected by a parasite (higher rectangles) or the number of parasite species an individual is infected by (lower rectangles). Inset: a map of the general region of the capture localities. Bipartite graphs were made using package 'bipartite' in the R environment.

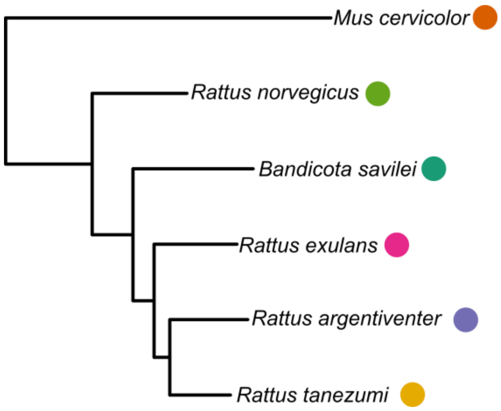

Figure S2. Phylogenetic tree. Colors match those of Fig. S1.

2. Controlling for network size and connectance in the analysis of network modularity.

The value of the modularity function M may be affected by network size or connectance. In our case, in each locality, the multi-species network was larger than each of the single-species networks and its connectance was lower (see Table 1 in the main text for exact number of individuals). To ensure that M of the multi-species network (M_m) was not affected by network size or connectance, we sub-sampled each of the multi-species networks 100 times as follows. In each of the 100 iterations we randomly chose n individuals, where n corresponds to the number of individuals in a single-species network to which comparison was made. We held the proportion of species constant. For example, in the original multi-species network in Mondolkiri, *Bandicota savilei* accounted for 62% of the individuals (23 of 37) and *Rattus tanezumi* for 38%. These proportions were kept for each sub-network.

Connectance of the sub-network was equalized to that of the single-species network by randomly removing edges from the sub-network. It was impossible to set the number of parasites equal to the original multi-species network because removal of individuals entailed removal of parasites. However, only sub-networks with at least six parasites were considered.

Under these conditions, we made four comparisons: *B. savilei* in Buriram; *B. savilei* and *R. tanezumi* in Mondolkiri; and *R. tanezumi* in Sihanouk (Fig. S3). We then calculated M for each of the 100 sub-networks in each comparison to produce a distribution of 100 values of M per locality. We examined where in the distribution M_m falls. If M_m does not fall beyond the 2.5% or 97.5% extremes, then our conclusions hold (i.e. a two-tailed permutation test). Below are the four histograms, with a red arrow indicating M_m. Only in Buriram was M_m affected by network size/connectance, but this can be discarded since the single-species network of *B. savilei* in Buriram was not significantly modular (see Table 1 in main text).

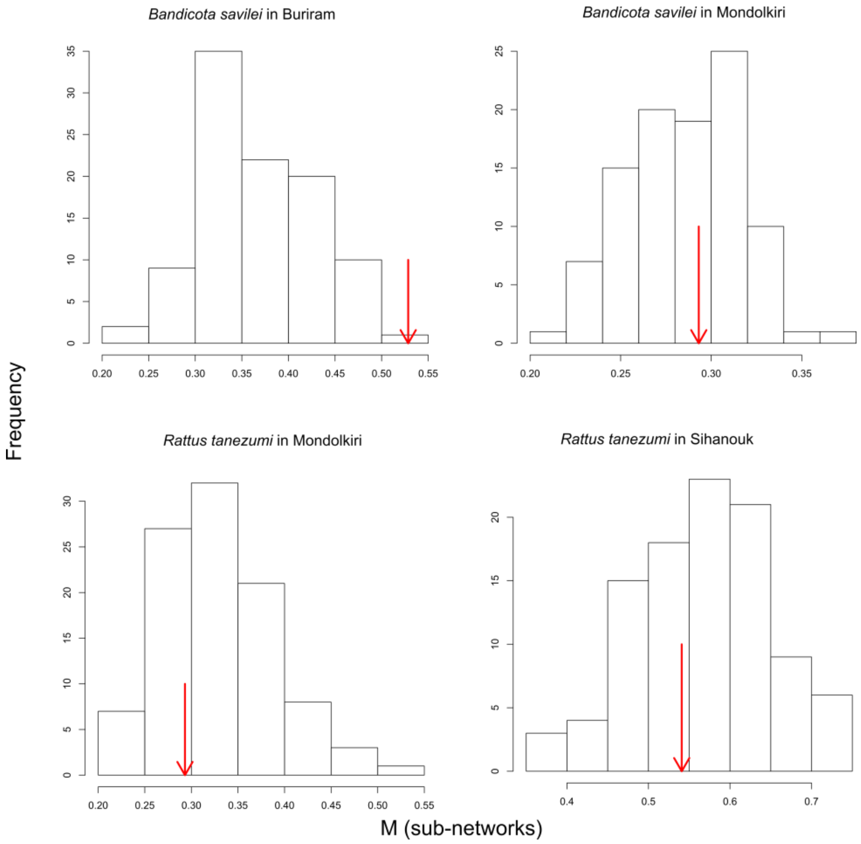

Figure S3. Comparisons of modularity values *M* for multi-species networks with the same size and connectance as single-species networks. Histograms show the distribution of randomized values of modularity, *M*. Red arrows indicate the observed value of M_m.

3. Multiple regression on distance matrices

We give an additional description of the multiple regression on distance matrices (MRM) using a graphical visualization (Fig. S4).

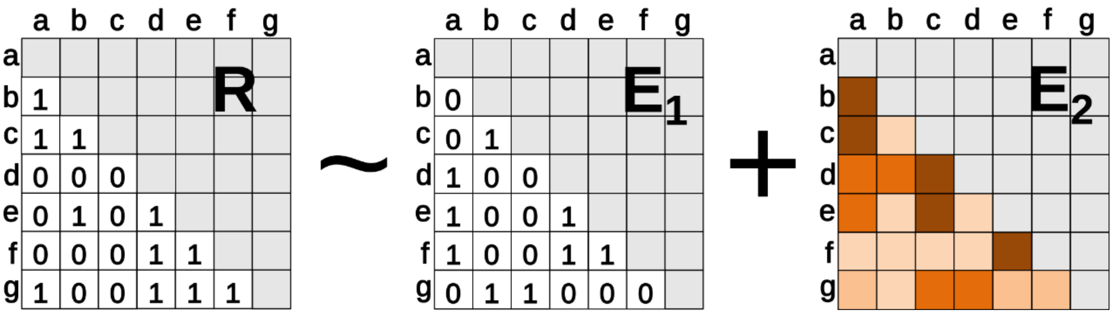

Figure S4. A visual description of the multiple regression on distance matrices (MRM) method. Rows and columns in each of the matrices are host individuals, depicted by lower-case letters a-g. Cell values in the response matrix, **R**, are 1 if two individuals are in the same module and 0 otherwise. Cell values in the explanatory matrix $\mathbf{E_1}$ are 1 if two individuals share the same characteristic (e.g. both are males), and 0 otherwise. $\mathbf{E_1}$ thus represents a categorical variable. Cell values in the explanatory matrix $\mathbf{E_2}$ are differences between pairs of individuals in a continuous characteristic (e.g. patristic distances or body mass). This is represented by the shade of brown (stronger the shade the larger the difference). $\mathbf{E_2}$ thus represents a categorical variable.

4. Construction of sub-TPNs.

Our goal was to compare TGI between a multi-species TPN and a single-species TPN (with >10 individuals) within the same locality. It is inappropriate, however, to compare networks of different sizes and connectance (i.e. the number of realized interactions divided by the number of possible ones). To control for different size and connectance while comparing multi-species TPNs to their respective single-species TPNs (within the same locality) we built 250 multi-species sub-TPNs by randomly sampling the original one to match the number of individuals of the single-species TPN. We kept the proportion of individuals of different species in the sub-TPN equal to that of the original multi-species TPN. For example, in the original multi-species network in Mondolkiri, *Bandicota savilei* accounted for 62% of the individuals (23 of 37) and *Rattus tanezumi* for 38%. These proportions were kept at each of the 250 sub-networks.

We also kept the connectance of the sub-TPNs constant to that of the original TPN. The connectance of the single-species TPN was always higher than that of the multi-species TPN (Table 1 in main text). Therefore, we built 250 single-species sub-TPNs by randomly removing edges from the original one to adjust for the connectance of the original multi-species network. The result was a set of 250 multi-species sub-TPNs and a set of 250 single-species sub-TPNs of equal size and connectance. For each of these 500 sub-TPNs we generated a distribution of 250 TGI values by randomly selecting individuals as starting points. We used the distribution of 250 mean TGI values (averaged for each sub-TPN) to examine differences between the single- and multi-species TPNs.

References

1. Herbreteau V, Jittapalapong S, Rerkamnuaychoke W, Chaval Y, Cosson J-F, et al. (2011) Protocols For Field And Laboratory Rodent Studies. 1st ed. Herbreteau V, Jittapalapong S, Rerkamnuaychoke W, Chaval Y, Cosson J-F, et al., editors Bangkok, Thailand: Kasetsart University Press. Available: http://www.ceropath.org/references/rodent_protocols_book.

2. Blasdell KR, Cosson JF, Chaval Y, Herbreteau V, Douangboupha B, et al. (2011) Rodent-borne Hantaviruses in Cambodia, Lao PDR, and Thailand. Ecohealth 8: 432–443.

3. Pakdeenarong N, Siribat P, Chaisiri K, Douangboupha B, Ribas A, et al. (2013) Helminth communities in murid rodents from southern and northern localities in Lao PDR: the role of habitat and season. J Helminthol: 1–8.

4. Paradis E, Claude J, Strimmer K (2004) APE: analyses of phylogenetics and evolution in R language. Bioinformatics 20: 289–290.

5. Pagès M, Chaval Y, Herbreteau V, Waengsothorn S, Cosson J-F, et al. (2010) Revisiting the taxonomy of the Rattini tribe: a phylogeny-based delimitation of species boundaries. BMC Evol Biol 10: 184. Available:

6. Palmeirim JM, Bordes F, Chaisiri K, Siribat P, Ribas A, et al. (2014) Helminth parasite species richness in rodents from Southeast Asia: role of host species and habitat. Parasitol Res.
