# Supplementary material for: Potential Parasite Transmission in Multi-Host Networks Based on Parasite Sharing
Source: PLoS One. 2015 Mar 6;10(3):e0117909. doi: 10.1371/journal.pone.0117909 (PMC4352066; doi:10.1371/journal.pone.0117909)
Supplement: S1 Table — (PDF) [file pone.0117909.s006.pdf]

Supporting Table S1. Comparison of models used for multi-model inference.

Models were obtained with a backwards stepwise regression starting from the global model and are ranked from the most to the least supported according to corrected Akaike information criteria (AICc). The global models contained all possible variables. Variables with missing cases (e.g. RSM in Mondokiri) or with no variation (e.g. when all individuals belonged to one sex) were excluded.

Model ranks	Model structure	Δ AICc	w_i
Buriram – multi-species			
1	EC~BM + Species	0	0.624
2	EC~BM + RSM + Species	1.61	0.279
3	EC~BM + RSM + Species + Sex	4.182	0.077
4	EC~Age + BM + RSM + Species + Sex	8.25	0.01
5 *	EC~Age + BM + Habitat + RSM + Species + Sex	8.25	0.01
6	EC~1 (Null)	25.788	0
Buriram – *Bandicota savilei*			
1	EC~BM	0	0.571
2	EC~1 (Null)	1.166	0.319
3	EC~BM + Sex	3.469	0.101
4 *	EC~Age + BM + Sex	8.081	0.01
Mondolkiri – multi-species			
1	EC~1 (Null)	0	0.503
2	EC~Species + Sex	0.642	0.365
3	EC~BM + Species + Sex	3.134	0.105
4	EC~Age + BM + Species + Sex	5.846	0.027
5 *	EC~Age + BM + Habitat + Species + Sex	13.222	0.001
Mondolkiri – *Bandicota savilei*			
1	EC~Age	0	0.397
2	EC~Age + Sex	0.619	0.291
3	EC~1 (Null)	1.393	0.198
4	EC~Age + BM + Sex	2.542	0.111
5 *	EC~Age + BM + Habitat + Sex	9.911	0.003
Mondolkiri – *Rattus tanezumi*			
1	EC~1 (Null)	0	0.599
2	EC~BM	1.918	0.229
3	EC~Age + BM	2.666	0.158
4	EC~Age + BM + Habitat	7.621	0.013

Model ranks	Model structure	Δ AICc	w_i
5 *	EC~Age + BM + Habitat + Sex	13.922	0.001
Sihanouk – multi-species			
1	EC~BM + RSM	0	0.667
2	EC~Age + BM + RSM	1.905	0.257
3	EC~Age + BM + RSM + Sex	4.695	0.064
4	EC~Age + BM + Habitat + RSM + Sex	8.429	0.01
5	EC~1 (Null)	11.202	0.002
6*	EC~Age + BM + Habitat + RSM + Species + Sex	18.219	0
Sihanouk – *Rattus exulans*			
1	EC~1 (Null)	0	0.767
2	EC~BM	2.555	0.214
3	EC~BM + Sex	7.45	0.018
4 *	EC~BM + RSM + Sex	14.618	0.001
Sihanouk – *Rattus tanezumi*			
1	EC~1 (Null)	0	0.776
2	EC~Sex	2.742	0.197
3	EC~RSM + Sex	6.844	0.025
4	EC~BM + RSM + Sex	12.416	0.002
5 *	EC~BM + Habitat + RSM + Sex	27.867	0

Δ AICc – difference in AICc between the current and best model; w_i – model probabilities.

Species – host species; BM – body mass; RSM – relative spleen mass to body mass (see Materials and Methods for details); EC – eigenvalue centrality; * – global model.
